# Incidence of Complicated Intrauterine Device Removal in a Large Health Care System

**DOI:** 10.1097/og9.0000000000000105

**Published:** 2025-08-14

**Authors:** Meaghan Coles, Dhriti Dedhia, Alejandro Alvarez, Shreya Sanghani, Matthew J. Blitz, Sharon Gerber

**Affiliations:** Northwell Health South Shore University Hospital, Department of Obstetrics and Gynecology, Bay Shore, the Donald and Barbara Zucker School of Medicine at Hofstra/Northwell, Hempstead, the Biostatistics Unit, Northwell Health Office of Academic Affairs, New Hyde Park, and the Northwell Health Feinstein Institutes for Medical Research, Manhasset, New York.

## Abstract

This retrospective study was designed to estimate the incidence of complicated intrauterine device (IUD) removals in a New York health care system between 2017 and 2020. *Complicated IUD removal* was defined as requiring instrumentation, ultrasound guidance, or removal in the operating room. Among 1,803 IUD removals, 205 (11.4%) were classified as complicated, with an incidence of 113.7 per 1,000 IUD removals (95% CI, 99–129.3). Age was the only demographic factor significantly associated with complicated removal (*P*<.01). Age and race and ethnicity were independently associated with operating room removal (*P*=.04 and *P*=.02, respectively). These results may support improved patient counseling on the potential risks of IUD removal.

## Abstract 2

Approximately 11% of intrauterine device removals in the general population require increased instrumentation or ultrasound guidance, with age, race and ethnicity, and primary language influencing incidence rates.

Long-acting reversible contraceptive use has increased steadily in the United States, rising from 6 insertions per 10,000 individuals in 2010 to 14.1 in 2020.^1^ Intrauterine devices (IUDs) are associated with lower failure rates, higher continuation rates, and higher patient satisfaction.^2^

Most IUD removals are routine, but complications can arise from missing strings, uterine wall embedment, perforation, or malposition. Management typically begins with a cytobrush attempt, followed by ultrasonography if unsuccessful.^3^ In some cases, further evaluation with abdominal X-ray, hysteroscopy, or laparoscopy is required.^4^ Few large studies report the incidence of complicated removals; two smaller studies found that approximately 80% of cases were uncomplicated.^4,5^

The primary objective of this study was to assess the incidence of complicated IUD removal within a large health care system. A secondary objective was to evaluate sociodemographic factors associated with complicated IUD removal.

## METHODS

We conducted a retrospective chart review of IUD removals in a large New York health care system between January 1, 2017, and December 31, 2020. Patients aged 15–55 years who underwent successful IUD removal were included, identified from inpatient and outpatient records using billing and diagnostic codes.

*Uncomplicated removals* were defined as office-based procedures with visible strings that did not require imaging or specialized instrumentation. *Complicated removals* involved ultrasound guidance, additional instrumentation, hysteroscopy, or surgical removal in an operating room.

The study protocol was reviewed by the Northwell Health IRB and deemed exempt. Two researchers independently reviewed all charts. Data were analyzed using χ^2^ and *t* tests in SAS software, with an alpha of 0.05. Race and ethnicity and primary language were included in the demographics used for statistical analysis to characterize the potential disparities in IUD complications for different populations, elucidating possible biases in access to and use of health care services. The sample size was based on available data in the electronic medical record system rather than formal power calculations.

## RESULTS

**Fig. 1.**
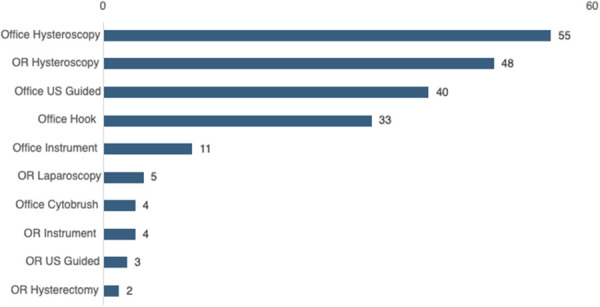
Types of complicated intrauterine device removal. Each encounter is mutually exclusive. OR, operating room; US, ultrasound.

Among 1,803 IUD removals, 205 (11.4%) were classified as complicated, with an incidence of 113.7 per 1,000 IUD removals (95.0% CI, 99–129.3). Age was the only demographic factor significantly associated with complicated removal (*P*=.02). Age and race and ethnicity were independently associated with operating room removal (*P*=.04 and *P*=.005, respectively). Among complicated removals, 143 (69.8%, 7.9% of total removals) occurred in the office and 62 (30.2%, 3.4% of total removals) required operating room intervention. Hysteroscopy was used in 50.2% of complicated removals, and ultrasonography was used in 21.0%. Demographic characteristics of the population are presented in Table 1, stratified by whether removal was complicated or uncomplicated (Fig. 1).

**Table 1.** Demographic Characteristics by Classification (Complicated vs Uncomplicated) and Setting (Office vs Operating Room) of Intrauterine Device Removal

Characteristic	Total Study Population (N=1,803)	IUD Removal					
		Uncomplicated (n=1,598)	Complicated (n=205)	*P*	Office (n=1,741)	Operating Room (n=62)	*P*
Age (y)	35.4	35.4	37.5	.02	35.6	37.9	.04
Race and ethnicity				.73			.005
Asian	104 (5.8)	88 (5.5)	16 (7.8)		96 (5.5)	8 (12.9)	
Black	243 (13.5)	215 (13.5)	28 (13.7)		228 (13.1)	15 (24.2)	
Hispanic	301 (16.7)	263 (16.5)	38 (18.5)		292 (16.8)	9 (14.5)	
None of the above	167 (9.3)	149 (9.3)	18 (8.8)		160 (9.2)	7 (11.2)	
Unknown	144 (8.0)	129 (8.1)	15 (7.3)		143 (8.2)	1 (1.6)	
White	844 (46.8)	754 (47.2)	90 (43.9)		822 (47.2)	22 (35.5)	
Preferred language				.03			.05
Spanish	101 (5.6)	83 (5.2)	18 (8.8)		94 (5.4)	7 (11.3)	
English	1,640 (91.0)	1,456 (91.1)	184 (89.8)		1,585 (91.0)	55 (88.7)	
None of the above	62 (3.4)	59 (3.7)	3 (1.5)		62 (3.6)	0 (0.0)	
BMI (kg/m^2^)				.61			.28
Lower than 25.0	490 (27.1)	431 (27.0)	59 (28.8)		469 (26.9)	21 (33.9)	
25.0–29.9	425 (23.6)	383 (24.0)	42 (20.5)		410 (23.6)	15 (24.2)	
30.0 or higher	397 (22.0)	347 (21.7)	50 (24.4)		374 (21.5)	23 (37.1)	
Unknown	491 (27.2)	437 (27.3)	54 (26.3)		488 (28.0)	3 (4.8)	

IUD, intrauterine device.

Data are mean±SD or n (column %) unless otherwise specified.

*Coles. Incidence of Complicated IUD Removals. O&G Open 2025*.

No significant associations were observed between race and ethnicity (*P*=.73) or body mass index (BMI, calculated as weight in kilograms divided by height in meters squared) (*P*=.61) and complicated IUD removal. However, Spanish-speaking patients had a higher rate of complicated removals (17.8%) compared with English-speaking patients (11.2%) and speakers of other languages (4.8%) (*P*=.03). In a secondary analysis comparing office removal with operating room removal, Spanish-speaking patients again were shown to have elevated rates of operating room removal (6.9%) compared with English-speaking patients (3.4%) (*P*=.05).

## DISCUSSION

In a large New York health care system, 11.4% of IUD removals were classified as complicated. As IUD use increases, counseling should address not only efficacy but also potential complications.^6^ Notably, 30.2% of complicated removals, and 3.4% of all removals, required operating room intervention, a risk likely unfamiliar to many patients.

Online narratives such as those on TikTok, where 40% of IUD-related content describes negative experiences, may contribute to patient concerns.^7^ However, our study showed that complicated removals are relatively uncommon in comparison. Recognizing this gap between perceived and actual complication rates is important for effective counseling. Previous studies suggest that higher BMI may increase the risk of difficult IUD removal.^8^ We, however, found no such association, supporting IUDs as a safe treatment option for individuals with obesity or related conditions such as polycystic ovarian syndrome.^9^

The higher rates of complicated and operating room removals among Spanish-speaking patients may reflect disparities in health care access, communication barriers, delayed follow-up, or clinician biases, all of which warrant further investigation. Finally, older age also was associated with increased risk of operating room removal, consistent with prior research.^9^

Strengths of the study include its large sample size and use of real-world clinical data. Limitations include its retrospective design, which relies on the accuracy of documentation, and the inclusion of only successful removals. Additionally, there were numerous patients excluded from the study because they never followed up in the health care system. This study provides key insights into the incidence of and factors associated with complicated IUD removals, informing patient counseling and clinical decision making.
